# Effects of COVID-19 government travel restrictions on mobility in a rural border area of Northern Thailand: A mobile phone tracking study

**DOI:** 10.1371/journal.pone.0245842

**Published:** 2021-02-03

**Authors:** Peter Haddawy, Saranath Lawpoolsri, Chaitawat Sa-ngamuang, Myat Su Yin, Thomas Barkowsky, Anuwat Wiratsudakul, Jaranit Kaewkungwal, Amnat Khamsiriwatchara, Patiwat Sa-angchai, Jetsumon Sattabongkot, Liwang Cui

**Affiliations:** 1 Faculty of ICT, Mahidol University, Nakhon Pathom, Thailand; 2 Bremen Spatial Cognition Center, University of Bremen, Bremen, Germany; 3 Department of Tropical Hygiene, Faculty of Tropical Medicine, Mahidol University, Bangkok, Thailand; 4 Center of Excellence for Biomedical and Public Health Informatics (BIOPHICS), Faculty of Tropical Medicine, Mahidol University, Bangkok, Thailand; 5 Faculty of Veterinary Science, Mahidol University, Nakhon Pathom, Thailand; 6 Mahidol Vivax Research Unit, Faculty of Tropical Medicine, Mahidol University, Bangkok, Thailand; 7 Division of Infectious Diseases and Internal Medicine, Department of Internal Medicine, University of South Florida, Tampa, Florida, United States of America; Ministry of Health and Sports, Myanmar, MYANMAR

## Abstract

**Background:**

Thailand is among the top five countries with effective COVID-19 transmission control. This study examines how news of presence of COVID-19 in Thailand, as well as varying levels of government restriction on movement, affected human mobility in a rural Thai population along the border with Myanmar.

**Methods:**

This study makes use of mobility data collected using a smartphone app. Between November 2019 and June 2020, four major events concerning information dissemination or government intervention give rise to five time intervals of analysis. Radius of gyration is used to analyze movement in each interval, and movement during government-imposed curfew. Human mobility network visualization is used to identify changes in travel patterns between main geographic locations of activity. Cross-border mobility analysis highlights potential for intervillage and intercountry disease transmission.

**Results:**

Inter-village and cross-border movement was common in the pre-COVID-19 period. Radius of gyration and cross-border trips decreased following news of the first imported cases. During the government lockdown period, radius of gyration was reduced by more than 90% and cross-border movement was mostly limited to short-distance trips. Human mobility was nearly back to normal after relaxation of the lockdown.

**Conclusions:**

This study provides insight into the impact of the government lockdown policy on an area with extremely low socio-economic status, poor healthcare resources, and highly active cross-border movement. The lockdown had a great impact on reducing individual mobility, including cross-border movement. The quick return to normal mobility after relaxation of the lockdown implies that close monitoring of disease should be continued to prevent a second wave.

## Introduction

Since the emergence of coronavirus disease 2019 (COVID-19) and the eventual declaration of a pandemic by the WHO, the primary measure used almost universally by countries worldwide to control transmission of the disease has been to limit human to human contact through social distancing and restrictions on movement. This has occurred through voluntary steps taken by citizens, as well as concrete government interventions. An important question to answer at this point is how effective the various measures have been at controlling movement that may contribute to disease transmission. A few studies have examined the relation between mobility and COVID-19 transmission in China [1, 2] and the United States [3, 4]. Those studies have been conducted at a macro scale and have primarily examined urban populations, which is important due to a number of factors such as high population density. But rural populations also warrant study, particularly due to the concern about lack of sufficient healthcare facilities to respond to serious COVID-19 outbreaks [5].

This study examines aspects of mobility relevant to transmission of COVID-19 in a rural population in Tak province, Thailand, located along the border with Myanmar. In addition to examining general mobility patterns, we examine dimensions of cross-border mobility. This study makes use of mobility data collected using a smartphone app installed on the phones of study participants. The mobility tracking was initially conducted to determine the cross-border mobility pattern as a potential risk factor for malaria infection along the border of Thailand [6]. Since collection of the mobility data began long before the COVID-19 pandemic and has continued to the present, the available data enables analysis of changes in mobility patterns from before the disease entered Thailand, through various government interventions to restrict movement, and into the phase of easing of these restrictions.

## Data and methods

### Study design

A cohort study was conducted nested within a large cohort study for malaria surveillance in Thailand, under the Southeast Asia-International Centers of Excellence for Malaria Research (ICEMR) project. This sub-cohort study was conducted from May 2019 to July 2020. All adults aged > 18 years who were enrolled in the original cohort were asked for voluntary participation in this sub-cohort study. As the aim of this sub-study was to describe the mobility pattern of people in the area, we planned to recruit all adults who own a working smartphone. According to our preliminary survey, people in the area generally own at least one mobile phone per house. However, a number of mobile phones were not eligible to be used in the study due to technical problems, such as full storage and unable to update the Android version.

### Study area

**Fig 1:**
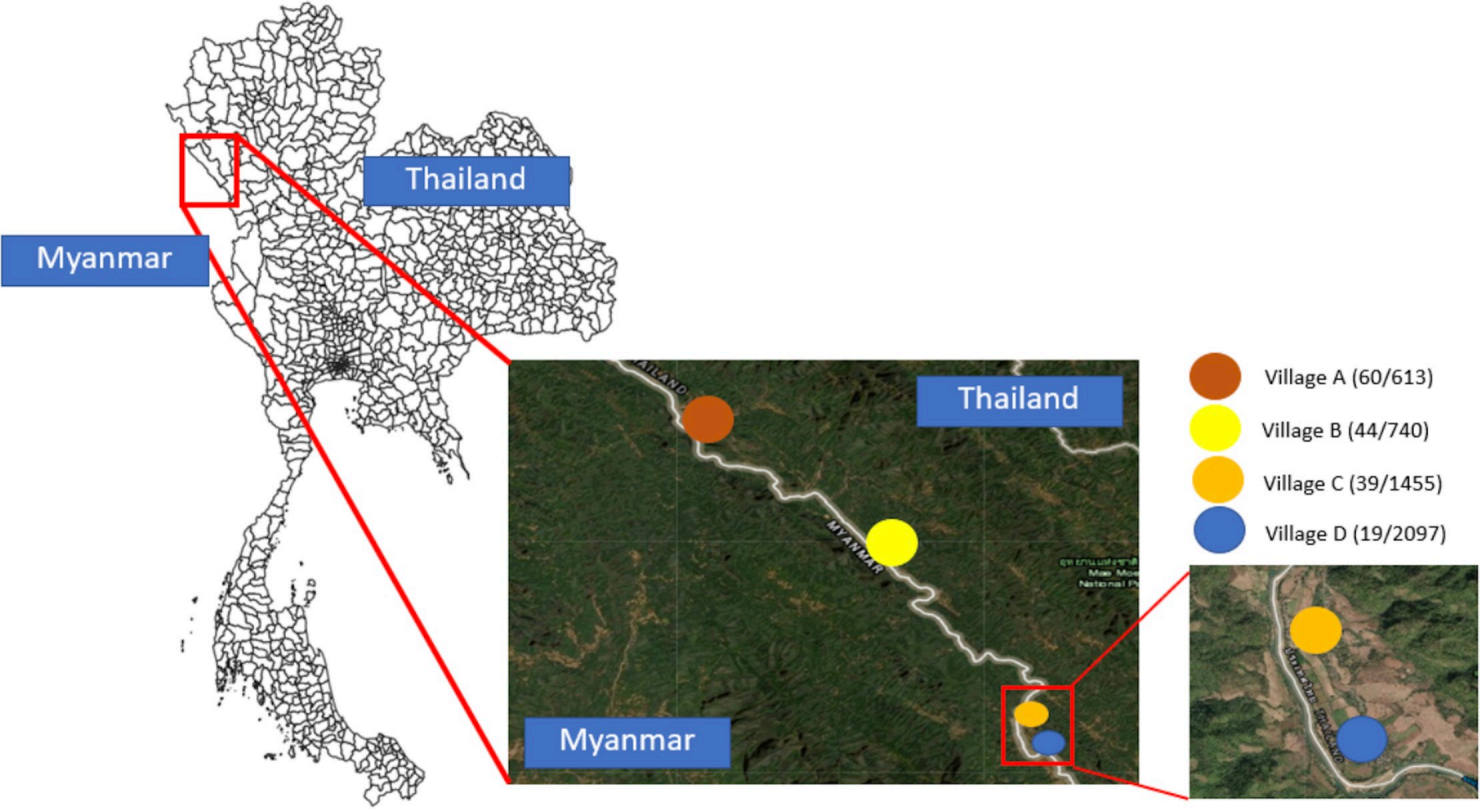
The study area consisting of four villages in Tha Song Yang district, Tak province of Thailand. The number of participants and total population of each village are shown next to the village name as (participants/population). The map in this figure was produced using ArcGIS version 10.4 (Esri, Redlands, CA, USA). Source of shapefile: United Nations Office for the Coordination of Humanitarian Affairs https://data.humdata.org/dataset/thailand-administrative-boundaries. The inset maps in the figure were created using an open-source map from https://landsatlook.usgs.gov/.

Fig 1 shows the main site of the study. Tha Song Yang district has a long border with Myanmar, with the Moei river acting as the natural boundary. Agriculture is a major part of the Tak economy, and many Karen migrants from Myanmar work in the agricultural sector there. The nearby Thai-Myanmar Friendship bridge across the river connects the Asian Highway between Thailand and Myanmar. However, people can also cross the river border by boat, without having to pass the government entry checkpoint.

### Selection of time intervals

We are interested in examining how news of the presence of COVID-19 in Thailand, as well as varying levels of government restriction on movement, affected dimensions of human mobility related to COVID-19 transmission. Between November 2019 and June 2020, there were four major such events relevant to mobility in our study area. The first COVID-19 case in Thailand was announced in the media on 13 January 2020 [7, 8]. Roughly two months later, a cumulative series of measures, which increasingly restricted mobility, were introduced over a period of two weeks. On 21 March 2020, the local government in Tak province announced the closure of the Friendship Bridge. On March 26, numerous provinces across the country introduced entry and exit restrictions and checkpoints were widely established on main roads to control inter district and inter sub-district travel [9]. On April 3, the central government introduced a nationwide curfew between 10pm and 4am [10]. This collection of restrictions was then in place from April 3 until travel restrictions were relaxed on May 2 and the curfew time was reduced to 11pm to 3am on May 3 [11].

These events give rise to five time intervals over which we compare mobility patterns:

- Interval 1, pre-COVID period: 1 November 2019 to 13 January 2020 (73 days)
- Interval 2, first publicized COVID cases: 14 January 2020 to 21 March 2020 (67 days)
- Interval 3, period of increasing restrictions: 22 March 2020 to 3 April 2020 (13 days)
- Interval 4, period of most restricted travel: 4 April 2020 to 3 May 2020 (30 days)
- Interval 5, relaxation of travel restrictions: 4 May 2020 to 2 June 2020 (31 days)

### Data collection

To gather mobility data, we developed an Android smartphone application that records and stores the phone’s geolocation on an hourly basis, using the Google Play Service [12]. To get the most accurate position, this service uses the phone's GPS, current cellular data and WiFi connection. The location obtained is specified through its geographic longitude and latitude, a timestamp, and the estimated horizontal accuracy in meters. The phone's location is recorded even when no phone signal is available, which is important in our study setting, where participants may travel through remote forested areas and into Myanmar. The application uploads the stored data to a server once an Internet connection is available. It is also designed to place very little demand on the battery or the phone’s storage space. Participants can control when they are tracked to protect their privacy in case they do not want to reveal their movement. The app includes a user ID, which associates the location data gathered from the phone with participant demographic information collected in the main cohort study, including sex, age, one of six occupations, and village of residence.

After receiving approval from the Ethics Committee of the Faculty of Tropical Medicine, Mahidol University (MUTM 2017-078-02), we recruited permanent residents aged over 18 from four villages in the study area (Fig 1). The participants were asked to install the application available in the Google Play Store. Project staff were responsible for the user ID registration and setting up WiFi hotspots each month for data transfer in villages where none were available. A data management application we developed was also used to evaluate the completeness of the collected data. Project staff checked on phones for which significant amounts of data were missing. This occurred in some cases when phones were old or in poor condition. There were 260 participants initially recruited in the study; 90 were excluded from the analysis because of missing data due to poor phone condition, leaving 162 participants. After a spatial accuracy of ±150 meters, the data trails off significantly, so we chose that value as the accuracy threshold to filter the data. This left 112,226 data points from November 1st, 2019 to June 2nd, 2020. After thresholding, there are 57, 49, 50, 55, 34 participants who have movement data in Intervals 1–5, respectively. Among all the participants, 21 are common to all five intervals.

## Data analyses

### Radius of gyration analysis

To characterize the distance traveled by each individual over a time period, we used the radius of gyration (RoG), a metric commonly used in analyses of human mobility [13, 14]. The RoG (r_g_) is defined as the root mean square distance from the centroid

$$r_{g}=\sqrt{\frac{1}{n}\sum_{i=1}^{n}{\left({\vec{p}}_{i}-{\vec{p}}_{centroid}\right)}^{2}}$$ (1)

where ${\vec{p}}_{i}$ is the i^th^ position for a participant and ${\vec{p}}_{centroid}$ is the center of mass of the participant calculated as

$${\vec{p}}_{centroid}=\frac{1}{n}\sum_{i=1}^{n}{\vec{p}}_{i}$$ (2)

The unit of measurement for RoG in this paper is meters. RoG was used to characterize the movement in each of the five temporal intervals of interest by computing the value for each participant over all his/her locations in each interval and summarizing median and interquartile range (IQR). To examine the impact of the curfew on mobility during the curfew hours, we computed each participant’s daily RoG during the curfew time (10pm - 4am) and the normal time (4am - 10pm) in each interval. We used the daily values since we are interested in movement within the time period of the curfew and not between curfew periods on different days. It is, for example, possible that someone could be in one location during curfew on one day and another during curfew on a second day but not move during the curfew time.

#### Human mobility network visualization

To visualize the main aggregate patterns of movement, human mobility network visualization was conducted for each time interval. First, main areas in which participants spent significant amounts of time were identified by clustering locations based on heat map visualization of the data. The intensity of travel between clusters was then represented with directed edges, resulting in a directed graph for each interval. The width of each edge was made proportional to the number of trips per participant per day in that interval, making edges comparable between intervals.

#### Cross-border and village boundary mobility analysis

A number of government measures involved restricting mobility across national and international borders. We thus examined movement across village boundaries, as well as movement across the border with Myanmar. We measured the number of short and long trips of each participant outside their village of residence and the proportion of time each participant spent outside their village. Since village shapefiles are not available in Thailand, we created a polygon to represent each village, based on a satellite image. The village polygons are shown in S1 Fig. A trip was defined as a set of points from the time of exiting the village polygon to the time of returning to it. The distance of a trip was defined as the maximum over all trip points of the distance from each trip point to the closest point on the polygon. The total time spent inside and outside the village was computed using the timestamps of each participant’s temporally ordered location points. The number of trips per participant per day was computed by taking the number of trips for each participant and averaging over participants and days in each interval. Next, we measured international cross-border movement by counting the number of trips from Thailand into Myanmar and the distance traveled into Myanmar for each trip. A trip was defined as a set of points from the time of crossing into Myanmar to the time of crossing back to Thailand. The distance into Myanmar of a trip was defined as the maximum distance from the border of all the trip points. The number of trips per participant per day was computed in the same way as for cross-village-boundary movement.

### Ethics statement

This study was approved by the Ethics Committee of the Faculty of Tropical Medicine, Mahidol University (MUTM 2017-078-02). All participants were individually informed about the study. Written informed consent was obtained from the participants. The participants could withdraw from the study at any time without any consequences.

## Results

Of the 162 participants, the majority were female (80%). The age of the participants ranged from 18 to 74, with mean 37 and standard deviation 12.3 years. 65% of the participants had Thai nationality, 31% Myanmar, and 4% others. There were six categories of occupation: 33% unemployed, 26% farmers, 23% laborers, 10% merchants, 2% public health personnel, and 6% others. Most of the participants were illiterate (61%), followed by secondary school (19%), primary school (16%), and others (4%).

### Radius of gyration

**Fig 2:**
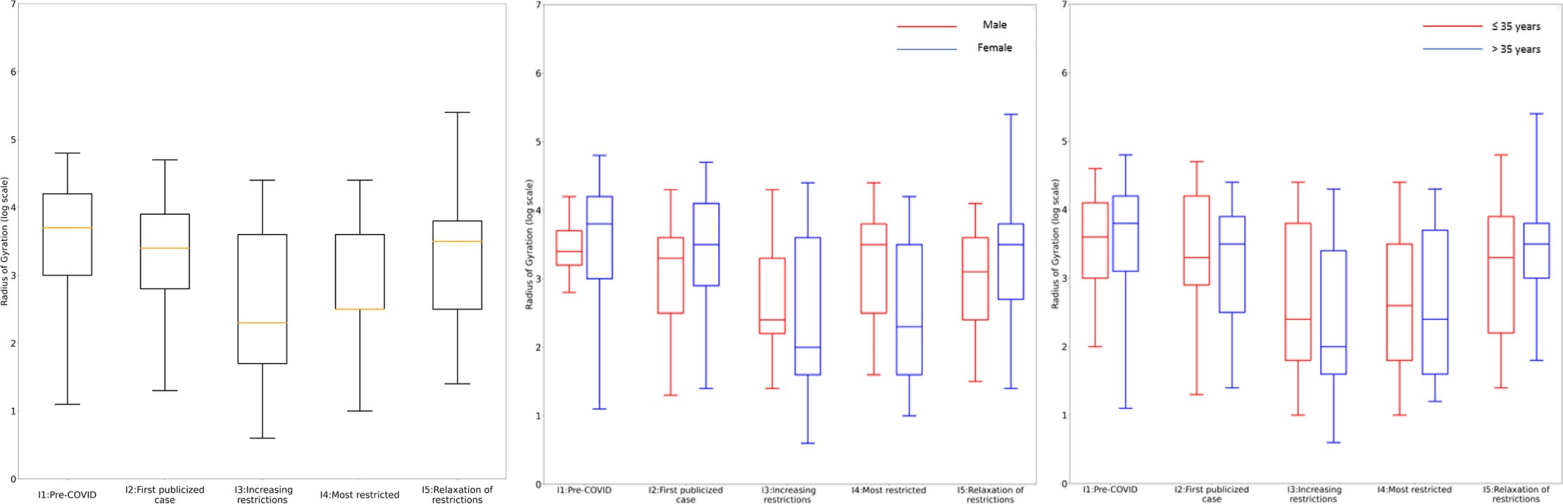
Overall RoG (a), stratified by Sex (b), and stratified by Age (c).

A box plot of the RoG values of the participants in all intervals is shown in Fig 2A. Interval 1 had by far the highest median RoG at 5.1 km. The median decreased to approximately half of this in Interval 2. Intervals 3 and 4 had similar extremely low median RoGs. In Interval 5 the value increased to even a bit higher than in Interval 2. The differences in median RoG values among the five intervals are all statistically significant (ANOVA F-ratio = 10.227, p < 0.001).

**Fig 3:**
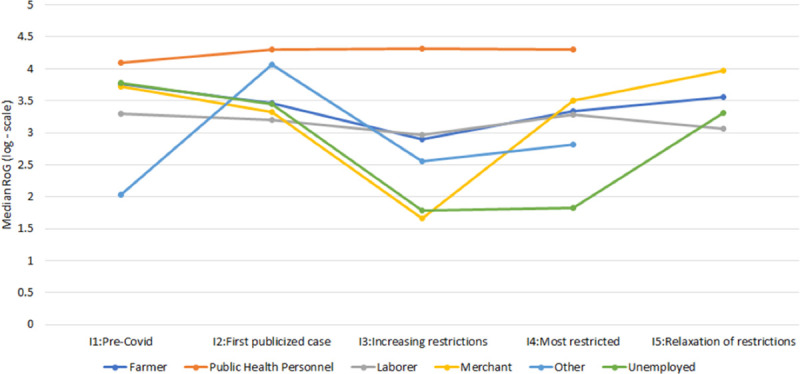
Median RoG values over the five intervals for each category of occupation (meters). No data is available in Interval 5 for public health personnel and other.

Fig 2B and 2C show box plots of the RoG values for the participants in each interval broken down by sex and age, respectively. Female participants had more movement than their male counterparts in Intervals 1, 2, and 5, while the male participants had more movement in Intervals 3 and 4, when travel restrictions were in place. The age group over 35 had more movement than the group under 35, except in Intervals 3 and 4. The changes in movement of both age groups followed the pattern of the entire cohort. In contrast, participants of differing occupations had rather different changes in movement over the intervals, as shown in Fig 3. In particular, the median values for public health personnel were much higher than for other occupations. The median values also increased in Intervals 2–4 over Interval 1. S4 Table in S1 File additionally shows the median, IQR, minimum, and maximum RoG values for the occupations.

**Fig 4:**
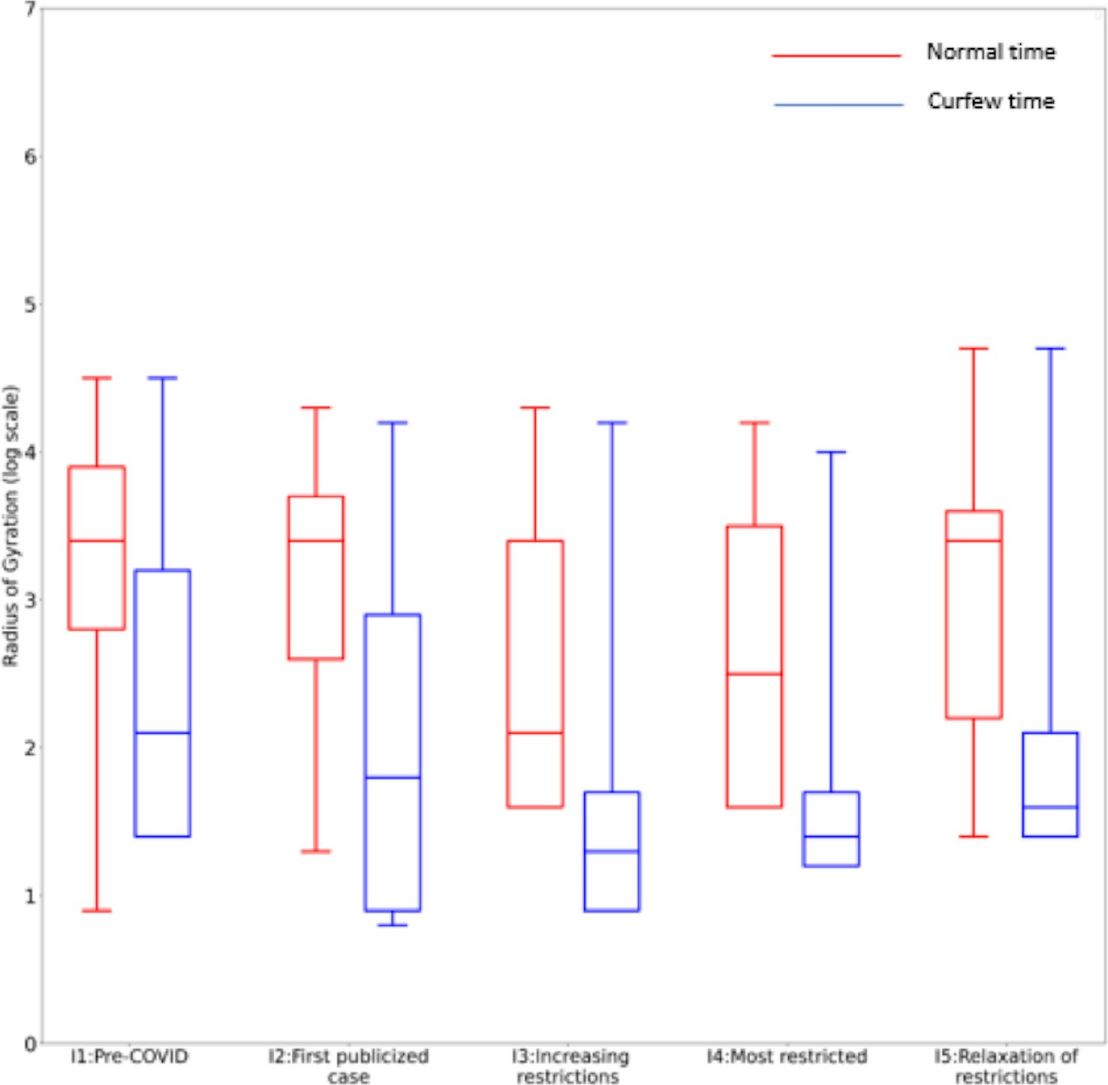
Daily RoG values during normal and curfew times in each interval (meters).

Arguably, the most stringent travel restriction introduced was the imposition of a curfew from 10pm to 4am at the beginning of Interval 3. Box plots of the *daily* RoG values of the participants in each interval during the curfew and normal times are shown in Fig 4. As would be expected, the RoG values were consistently lower during nighttime hours than during the daytime. During the curfew hours, the RoG declined to markedly low values in Intervals 3 and 4, showing the impact of the restriction. The pattern of RoG values during normal hours was similar to that over the entire interval except that the value in Interval 1 was much lower. This is not surprising since these are daily RoG values. The differences between the log-transformed curfew time RoGs were significant using one-way ANOVA with an F-ratio of 8.31633 and p < 0.00001. For the log-transformed normal time RoGs, the differences were also significant with F-ratio of 9.66776 and p < 0.00001. S6–S13 Tables in S1 File show the curfew and normal time RoG values stratified by sex, age, and occupation. Analysis by occupation shows that the RoG values for government officers had a markedly different pattern, being higher in Intervals 2–4 than in Interval 1.

### Human mobility network

**Fig 5:**
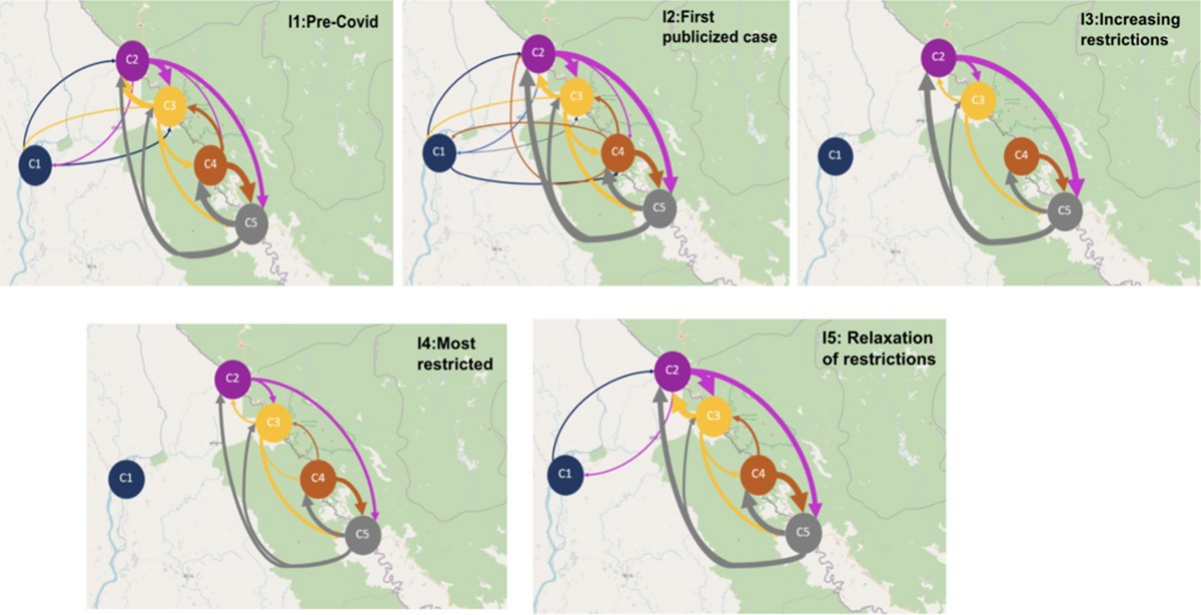
Human mobility network visualization for Intervals 1–5. Each node represents an activity cluster. Each edge denotes human movement in the direction of the arrow. The thickness of the edges is proportional to the number of trips between the nodes. Cluster C1 corresponds to a small town in Myanmar; C2 corresponds to village A, C3 corresponds to village B; C4 covers villages C and D; and C5 corresponds to the district capital. The maps in this figure were created using an open-source map from https://landsatlook.usgs.gov/.

Heatmap visualization identified five clusters of data points, as shown in Fig 5. Cluster C1 covers points in Myanmar, while the remaining clusters are in Thailand. Clusters C2, C3, and C4 cover the four villages of the participants, with cluster C4 covering two adjacent villages. Cluster C5 covers the capital of Tha Song Yang district. Intervals 1 and 2 had the most travel, with the most intensive travel between three pairs of clusters: C2-C3, C2-C5, and C4-C5. In Interval 3, travel was significantly curtailed, and all travel to and from cluster C1 in Myanmar had ceased, although as the cross-border analysis below will show, some travel to other locations in Myanmar remained. Interval 4 had an even greater reduction in travel. The presence of data points in cluster C1 during Intervals 3 and 4 indicates that some participants traveled to that location in Myanmar in earlier intervals and stayed there. In Interval 5, we see a resumption of travel, including that to and from cluster C1 in Myanmar.

### Cross-border and village boundary mobility analysis

**Fig 6:**
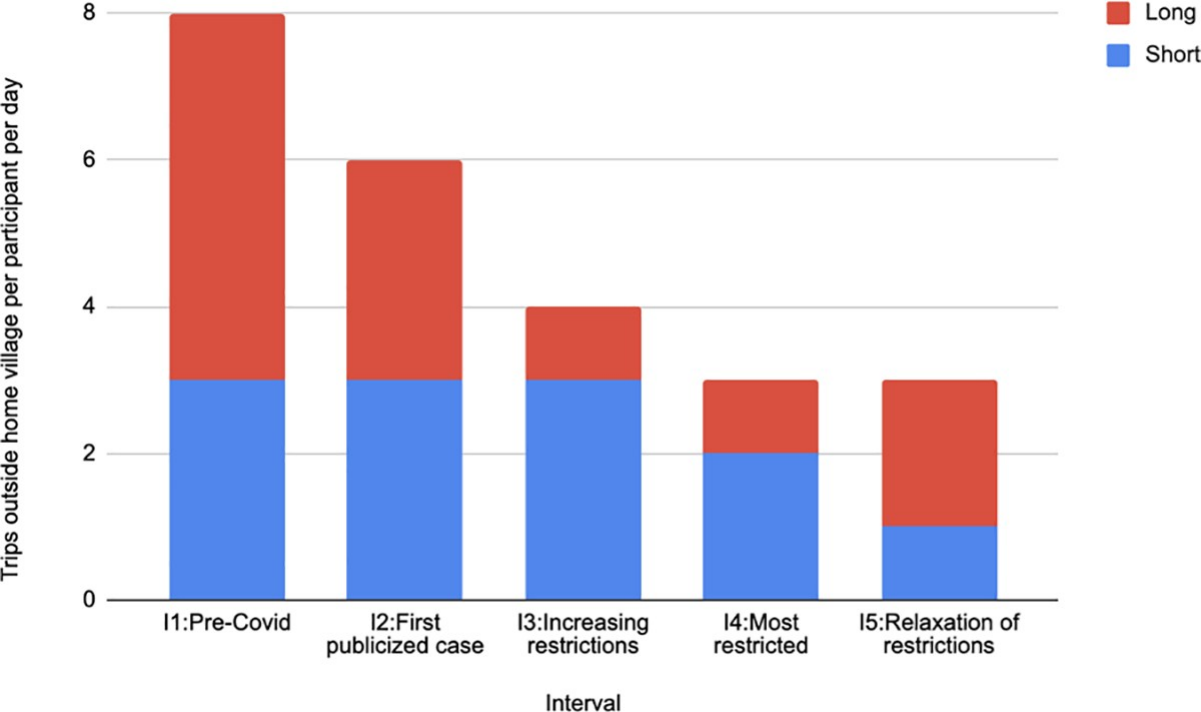
Number of trips outside the home village per participant per day in each interval, broken down into long and short trips. Short trips are defined as those for which the maximum distance from the village boundary is less than 500 meters and long trips are those greater than 500 meters.

Another interesting aspect of mobility is the extent of travel outside each participant’s home village during each interval. To determine this, we categorized the participant locations over time into those *inside* the village and those *outside* the village. The number of long and short trips in each interval are shown in Fig 6. The number of trips was computed for each participant and averaged over participants and days in each interval. As restrictions tightened, we saw a decrease in the number of trips, and particularly in the number of long trips. Comparing the number of trips between Interval 1, the least restricted, and Interval 4, the most restricted, we saw a decrease in short trips of 32% and a decrease in long trips of 70%. Once restrictions were relaxed in Interval 5, the number of long trips increased. S19 Table in S1 File shows additionally the percentage time spent outside the home village, which displayed no particular pattern. S20 Table in S1 File shows the data broken down by village, where we see quite different patterns of mobility among the four villages.

**Fig 7:**
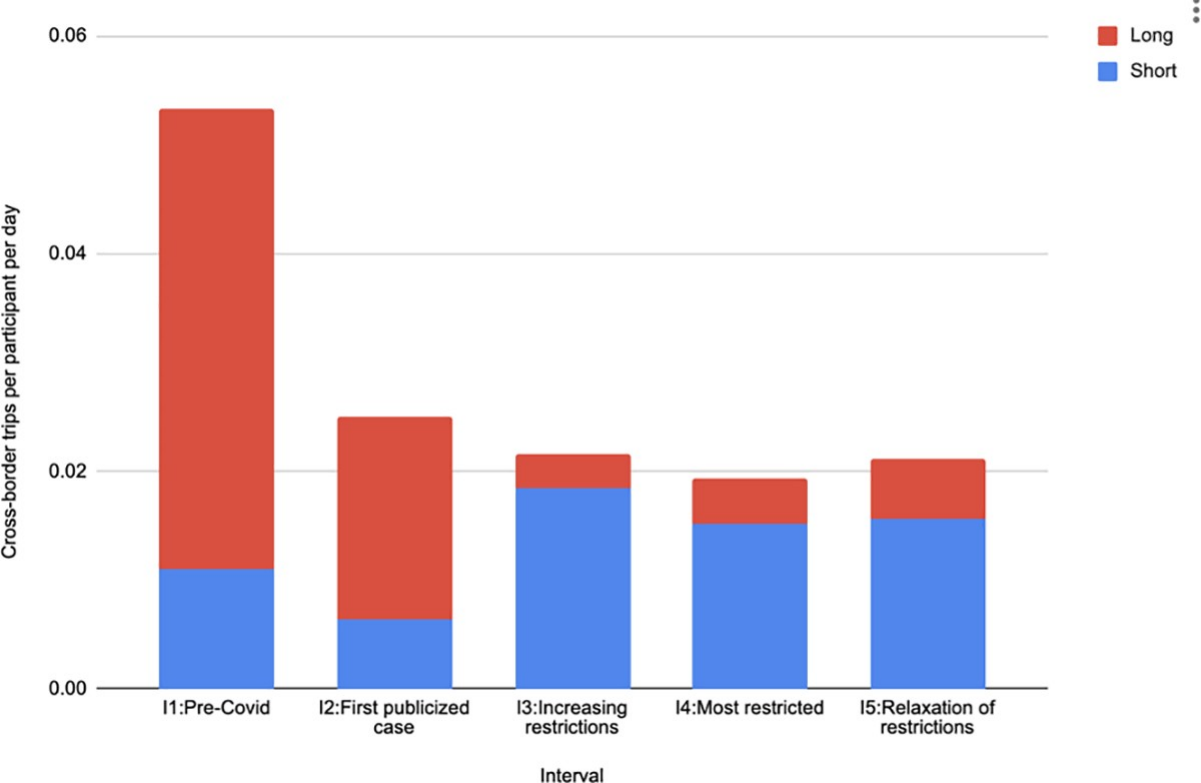
Number of cross-border trips per participant per day in each interval, broken down into long and short trips. Short trips are defined as those for which the maximum distance from the border is less than 500 meters and long trips are those greater than 500 meters.

Next, we measured international cross-border movement from Thailand into Myanmar. Fig 7 shows the number of cross-border trips per participant, broken downs into long and short trips. The number of trips in Interval 1 was roughly double that in the other intervals, including Interval 5. The proportion of long versus short trips changed over time. The proportion of long trips was larger in Intervals 1 and 2, while the proportion of short trips was larger in Intervals 3–5. The median distance (S14 Table in S1 File) reached its highest level in Interval 2 and dropped off significantly in the remaining intervals. While the network analysis (Fig 5) showed no travel to cluster C1 in Myanmar during Intervals 3 and 4, Fig 7 shows cross-border movement in those intervals due primarily to short trips and to a few long trips to locations other than cluster C1.

S15–S18 Tables in S1 File contain a number of additional measures of cross-border travel, including stratification by sex, age, occupation and home village. In general, female participants made more cross-border trips than male participants and participants ≤ 35 years old made more cross border trips than participants >35 years old. Among occupations, in Interval 1 the most short trips were made by laborers, followed by unemployed, and farmers, while the most long trips were made by farmers, followed by unemployed. In the most restricted interval (Interval 4), the most short trips were made by laborers, followed by unemployed, with farmers making almost no short trips. In that same interval, long trips were only made by farmers and unemployed.

## Discussion

Human mobility is a critical factor in determining disease transmission, particularly for COVID-19 [1]. Traditional public health interventions may not be sufficient to contain new emerging diseases for which no vaccine is available. Community lockdown and travel restrictions have been shown to be effective to control the transmission of such diseases, including Severe Acute Respiratory Syndrome (SARS), Ebola, pandemic Swine flu (H1N1), and the current pandemic COVID-19 [15–18]. However, community lockdown requires great effort at the government and community levels; and therefore, the effectiveness can vary across different countries and settings [2–4]. Thailand, which is among the top five countries with effective COVID-19 transmission control, has shown a dramatic reduction of cases after the lockdown policy was implemented [19]. In this study, the direct impact of the lockdown policy on human mobility was assessed in a rural border area of Thailand.

Population movement patterns are generally different between rural and urban areas, due to geographical condition, lifestyle, and occupation of people in the area. This can create different transmission patterns of disease. In the study area, people are mostly of Karen origin, a minority ethnic group along the Thai-Myanmar border. The main occupations are agriculture and general labor. Inter-village and cross-border movements are commonly observed in the area [20], as also indicated by our findings during the pre-COVID-19 period (Interval 1).

Thailand reported the first imported COVID-19 case on January 14th, 2020. With a tremendous effort to control the disease, only a few imported cases were reported each day. Although COVID-19 cases were mostly confined to Bangkok, there was public concern regarding prevention of disease throughout the country. People tended to avoid unnecessary social contact and movement. This preventive behavior was also observed in this study, where the RoG and cross-border trips decreased during Interval 2. Clusters of COVID-19 local transmission were initially observed at the end of March, with cases reported in different provinces across the country. Local authorities started to strengthen control measures, including closure of cross-border bridges and establishment of immigration checkpoints [9]. The percentage of participants who took cross-border trips greatly reduced from 38.8% to 9.8% after the official closure of the Friendship Bridge.

The reduction in population mobility was also observed during the national lockdown period (Interval 4). Previous studies conducted in metropolitan areas of China and the US have shown that government lockdown could reduce individual mobility up to 50% [2–4]. However, the impact of government lockdown was much greater in this study area in Thailand; the RoG was reduced by more than 90%. Although there was no COVID-19 case reported in the area, public trust is an important factor that determines people’s compliance with disease prevention measures [21]. Interestingly, public health personnel had relatively high RoG during the lockdown period. The movement was also observed during the curfew time, compared with other occupations. Local health officers, including village health volunteers, played an important role in COVID-19 transmission control in Thailand. Public health officials usually collaborate closely with village health volunteers to support government health policy and to build community trust. This system has greatly contributed to the success in disease prevention and control of the country [22].

Although lockdown policy is a powerful and effective measure to control COVID-19 [1–4], it has a significant impact on the economy [23]. In Thailand, disease transmission was well controlled after one month of the national lockdown. Later on, the travel restriction was relaxed to allow economic growth. This study found that human mobility was nearly back to normal after relaxation of the lockdown period (Interval 5). In fact, examining the RoG within Interval 5, we find that mobility rebounded already in the first two weeks. Therefore, close monitoring of disease outbreak should be continued to prevent a second wave of COVID-19 transmission. A previous study has shown that a second wave of epidemic is likely to occur after relaxation of strict intervention [24].

Mobility data can be captured using different methods, including interviews and diary keeping [25], GPS loggers [26], and mobile phone calling records [27]. These methods have important limitations such as limited precision in time and space (interviews and diaries, calling records), cost (GPS loggers), and inability to monitor individual movement patterns (calling records). By tracking individual mobile phones and linking the phones to individuals’ demographic data, this study was able to gather information on individual mobility with high precision and at low cost.

This study used a variety of analysis techniques to examine dimensions of mobility relevant to COVID-19 transmission. Radius of gyration indicates the characteristic distance travelled by a person during a time period. Adjusting the time period enabled analysis of two different aspects of mobility—motion throughout an interval and daily motion inside and outside of curfew periods. Network analysis identifies geographic locations of high activity, as well as travel patterns between them, indicating likely routes of disease transmission. Finally, the cross-border mobility analysis highlights the potential for intercountry disease transmission.

This study was conducted in a small area along the Thai-Myanmar border, and not all the population participated in the mobility tracking. Findings may not be generalizable to the entire country. However, the study provides insight into the impact of the COVID-19 epidemic and the government lockdown policy on an area with extremely low socio-economic status, poor healthcare resources, and highly active cross-border movement [20].

## Conclusion

Thailand is among the top countries with effective COVID-19 transmission control. The government lockdown policy had a great impact on reducing individual mobility, including cross-border movement. Local government and healthcare personnel had been active during the lockdown period. This local support potentially contributed to the success of the government lockdown policy to prevent the COVID-19 epidemic in the country.

## Supporting information

S1 File (DOCX)

S1 Fig Polygons of the four villages in the study area. (TIF)
