# Supplementary material for: Effects of COVID-19 government travel restrictions on mobility in a rural border area of Northern Thailand: A mobile phone tracking study
Source: PLoS One. 2021 Feb 3;16(2):e0245842. doi: 10.1371/journal.pone.0245842 (PMC7857734; doi:10.1371/journal.pone.0245842)
Supplement: S1 File — (DOCX) [file pone.0245842.s001.docx]

**S1 File.**

# Radius of gyration analysis

Table S-1. Overall RoG (in meters)

|  | **Minimum** | **Q1** | **Median** | **Q3** | **Maximum** |
| --- | --- | --- | --- | --- | --- |
|  |  |  |  |  |  |
| I1:Pre-Covid | 11 | 1004 | 5,093 | 14240 | 56,905 |
| I2:First publicized case | 22 | 679 | 2,403 | 8263 | 49,823 |
| I3:Increasing restrictions | 4 | 46 | 180 | 3641 | 24,690 |
| I4:Most restricted | 9 | 324 | 324 | 3705 | 22,535 |
| I5:Relaxation of restrictions | 24 | 293 | 2,933 | 6325 | 230,681 |

Table S-2. RoG (in meters) - by Sex

|  | **Minimum** | **Q1** | **Median** | **Q3** | **Maximum** |
| --- | --- | --- | --- | --- | --- |
|  |  |  |  |  |  |
| **Male** |  |  |  |  |  |
| I1:Pre-COVID | 589 | 1590 | 2678 | 5474 | 17,033 |
| I2:First publicized case | 22 | 287 | 2230 | 3733 | 19,848 |
| I3:Increasing restrictions | 22 | 157 | 227 | 1858 | 20,603 |
| I4:Most restricted | 36 | 282 | 2949 | 6284 | 22,535 |
| I5:Relaxation of restrictions | 32 | 240 | 1396 | 4341 | 11,665 |
|  |  |  |  |  |  |
| **Female** |  |  |  |  |  |
| I1:Pre-COVID | 11 | 996 | 6752 | 15973 | 56,905 |
| I2:First publicized case | 23 | 790 | 2952 | 13199 | 49,823 |
| I3:Increasing restrictions | 4 | 41 | 110 | 4354 | 24,690 |
| I4:Most restricted | 9 | 40 | 211 | 3455 | 17,577 |
| I5:Relaxation of restrictions | 24 | 490 | 3115 | 6893 | 230,681 |

Table S-3. RoG (in meters) - by Age

|  | **Minimum** | **Q1** | **Median** | **Q3** | **Maximum** |
| --- | --- | --- | --- | --- | --- |
|  |  |  |  |  |  |
| ***<= 35*** |  |  |  |  |  |
| I1:Pre-COVID | 92 | 1004 | 3562 | 13159 | 36,240 |
| I2:First publicized case | 22 | 788 | 2086 | 14189 | 49,823 |
| I3:Increasing restrictions | 10 | 64 | 229 | 6589 | 24,690 |
| I4:Most restricted | 9 | 63 | 389 | 3346 | 22,535 |
| I5:Relaxation of restrictions | 24 | 151 | 2022 | 7110 | 68,294 |
|  |  |  |  |  |  |
| ***>35*** |  |  |  |  |  |
| I1:Pre-COVID | 11 | 1317 | 6435 | 16326 | 56,905 |
| I2:First publicized case | 23 | 306 | 3389 | 7984 | 26,686 |
| I3:Increasing restrictions | 4 | 41 | 112 | 2553 | 20,284 |
| I4:Most restricted | 14 | 43 | 245 | 5087 | 19,756 |
| I5:Relaxation of restrictions | 68 | 1108 | 2933 | 5742 | 230,681 |

Table S-4. RoG (in meters) - by Occupation

|  | **Minimum** | **Q1** | **Median** | **Q3** | **Maximum** |
| --- | --- | --- | --- | --- | --- |
|  |  |  |  |  |  |
| ***Farmer*** |  |  |  |  |  |
| I1:Pre-COVID | 401 | 2519 | 5862 | 11737 | 18372 |
| I2:First publicized case | 247 | 886 | 2873 | 7170 | 49823 |
| I3:Increasing restrictions | 25 | 192 | 779 | 3958 | 24690 |
| I4:Most restricted | 14 | 104 | 2188 | 7375 | 15017 |
| I5:Relaxation of restrictions | 116 | 2004 | 3584 | 4446 | 68294 |
|  |  |  |  |  |  |
| ***Public health personnel*** |  |  |  |  |  |
| I1:Pre-COVID | 7,792 | 10102 | 12412 | 14722 | 17,033 |
| I2:First publicized case | 19,848 | 19847 | 19847 | 19847 | 19,848 |
| I3:Increasing restrictions | 20,284 | 20284 | 20284 | 20284 | 20,284 |
| I4:Most restricted | 19,756 | 19755 | 19755 | 19755 | 19,756 |
| I5:Relaxation of restrictions | N/A | N/A | N/A | N/A | N/A |
|  |  |  |  |  |  |
| ***Laborer*** |  |  |  |  |  |
| I1:Pre-COVID | 285 | 689 | 1960 | 12840 | 43,530 |
| I2:First publicized case | 207 | 772 | 1587 | 2686 | 14,322 |
| I3:Increasing restrictions | 58 | 772 | 927 | 2686 | 20,603 |
| I4:Most restricted | 36 | 316 | 1893 | 6531 | 22,535 |
| I5:Relaxation of restrictions | 83 | 397 | 1164 | 8946 | 11,665 |
|  |  |  |  |  |  |
| ***Merchant*** |  |  |  |  |  |
| I1:Pre-COVID | 950 | 1795 | 5214 | 12670 | 29,738 |
| I2:First publicized case | 22 | 31 | 2106 | 7595 | 17,850 |
| I3:Increasing restrictions | 4 | 13 | 45 | 302 | 11,676 |
| I4:Most restricted | 9 | 2715 | 3182 | 3737 | 10,197 |
| I5:Relaxation of restrictions | 32 | 4644 | 9257 | 13869 | 18,482 |
|  |  |  |  |  |  |
| ***Other*** |  |  |  |  |  |
| I1:Pre-COVID | 107 | 107 | 107 | 107 | 107 |
| I2:First publicized case | 3096 | 7383 | 11670 | 15958 | 20245 |
| I3:Increasing restrictions | 106 | 231 | 358 | 9664 | 18971 |
| I4:Most restricted | 211 | 434 | 657 | 881 | 1104 |
| I5:Relaxation of restrictions | N/A | N/A | N/A | N/A | N/A |
|  |  |  |  |  |  |
| ***Unemployed*** |  |  |  |  |  |
| I1:Pre-COVID | 11 | 1264 | 5922 | 21363 | 56,905 |
| I2:First publicized case | 23 | 182 | 2808 | 12209 | 26,987 |
| I3:Increasing restrictions | 5 | 40 | 61 | 343 | 18,522 |
| I4:Most restricted | 16 | 37 | 66 | 324 | 17,577 |
| I5:Relaxation of restrictions | 24 | 84 | 2027 | 6059 | 230,681 |

# Curfew & Normal time RoG

Table S-5. Normal time and curfew time RoG (in meters)

|  | **Minimum** | **Q1** | **Median** | **Q3** | **Maximum** |
| --- | --- | --- | --- | --- | --- |
|  |  |  |  |  |  |
| **Normal** |  |  |  |  |  |
| I1:Pre-Covid | 9 | 678 | 2380 | 7532 | 35,004 |
| I2:First publicized case | 21 | 375 | 2252 | 5362 | 19,945 |
| I3:Increasing restrictions | 0 | 40 | 118 | 2489 | 19,803 |
| I4:Most restricted | 0 | 39 | 297 | 3229 | 17,580 |
| I5:Relaxation of restrictions | 23 | 146 | 2247 | 4199 | 49,540 |
|  |  |  |  |  |  |
| **Curfew** |  |  |  |  |  |
| I1:Pre-Covid | 0 | 28 | 132 | 1514 | 31,081.07 |
| I2:First publicized case | 6 | 8 | 63 | 834 | 15,771.07 |
| I3:Increasing restrictions | 0 | 8 | 19 | 48 | 14,658.74 |
| I4:Most restricted | 0 | 15 | 26 | 53 | 9,169.89 |
| I5:Relaxation of restrictions | 0 | 23 | 44 | 136 | 56,059.82 |

Table S-6. Curfew time RoG (in meters) - by Sex

|  | **Minimum** | **Q1** | **Median** | **Q3** | **Maximum** |
| --- | --- | --- | --- | --- | --- |
|  |  |  |  |  |  |
| ***Male*** |  |  |  |  |  |
| I1:Pre-Covid | N/A | 97.15 | 479.18 | 1220.8 | 8,235 |
| I2:First publicized case | 16 | 27.39 | 71.71 | 159.71 | 11,459 |
| I3:Increasing restrictions | 6 | 11.76 | 25.92 | 56.2 | 14,659 |
| I4:Most restricted | N/A | 17.03 | 25.99 | 99.38 | 9,170 |
| I5:Relaxation of restrictions | 24 | 30.59 | 56.6 | 123.83 | 3,052 |
|  |  |  |  |  |  |
| ***Female*** |  |  |  |  |  |
| I1:Pre-Covid | 0 | 97.15 | 479.18 | 1220.8 | 8,235 |
| I2:First publicized case | 16 | 27.39 | 71.71 | 159.71 | 11,459 |
| I3:Increasing restrictions | 6 | 11.76 | 25.92 | 56.2 | 14,659 |
| I4:Most restricted | 0 | 17.03 | 25.99 | 99.38 | 9,170 |
| I5:Relaxation of restrictions | 24 | 30.59 | 56.6 | 123.83 | 3,052 |

Table S-7. Curfew time RoG (in meters) - by Age

|  | **Minimum** | **Q1** | **Median** | **Q3** | **Maximum** |
| --- | --- | --- | --- | --- | --- |
|  |  |  |  |  |  |
| ***<= 35*** |  |  |  |  |  |
| I1:Pre-Covid | 10 | 30.68 | 132.43 | 1165.57 | 31,081 |
| I2:First publicized case | 9 | 19.89 | 71.71 | 834.18 | 15,771 |
| I3:Increasing restrictions | 0 | 8.52 | 24.34 | 60.3 | 14,659 |
| I4:Most restricted | 0 | 15.44 | 28 | 52.65 | 288 |
| I5:Relaxation of restrictions | 0 | 24.63 | 36.09 | 128.12 | 4,737 |
|  |  |  |  |  |  |
| ***>35*** |  |  |  |  |  |
| I1:Pre-Covid | 0 | 24.07 | 165.83 | 5008.99 | 22,346 |
| I2:First publicized case | 6 | 17.05 | 62.59 | 511.02 | 11,459 |
| I3:Increasing restrictions | 0 | 10.16 | 19.72 | 48.76 | 10,550 |
| I4:Most restricted | 0 | 14.44 | 26.52 | 79.87 | 9,170 |
| I5:Relaxation of restrictions | 0 | 22.12 | 51.1 | 109.05 | 56,060 |

Table S-8. Curfew time RoG (in meters) - by Occupation

|  | **Minimum** | **Q1** | **Median** | **Q3** | **Maximum** |
| --- | --- | --- | --- | --- | --- |
|  |  |  |  |  |  |
| ***Farmer*** |  |  |  |  |  |
| I1:Pre-Covid | 0 | 26 | 49 | 1310 | 9770 |
| I2:First publicized case | 16 | 46 | 88 | 957 | 15771 |
| I3:Increasing restrictions | 0 | 13 | 57 | 118 | 4892 |
| I4:Most restricted | 0 | 12 | 23 | 50 | 251 |
| I5:Relaxation of restrictions | 12 | 36 | 77 | 152 | 1466 |
|  |  |  |  |  |  |
| ***Public health personnel*** |  |  |  |  |  |
| I1:Pre-Covid | 3034 | 4335 | 5635 | 6935 | 8235 |
| I2:First publicized case | 11459 | 11459 | 11459 | 11459 | 11459 |
| I3:Increasing restrictions | 10550 | 10550 | 10550 | 10550 | 10550 |
| I4:Most restricted | 9170 | 9170 | 9170 | 9170 | 9170 |
| I5:Relaxation of restrictions | N/A | N/A | N/A | N/A | N/A |
|  |  |  |  |  |  |
| ***Laborer*** |  |  |  |  |  |
| I1:Pre-Covid | 6 | 45 | 331 | 710 | 22346 |
| I2:First publicized case | 9 | 49 | 76 | 288 | 4581 |
| I3:Increasing restrictions | 6 | 22 | 39 | 93 | 14659 |
| I4:Most restricted | 3 | 18 | 25 | 94 | 3986 |
| I5:Relaxation of restrictions | 27 | 44 | 57 | 60 | 3052 |
|  |  |  |  |  |  |
| ***Merchant*** |  |  |  |  |  |
| I1:Pre-Covid | 10 | 42 | 335 | 4566 | 6328 |
| I2:First publicized case | 10 | 14 | 18 | 582 | 2269 |
| I3:Increasing restrictions | 1 | 3 | 9 | 15 | 34 |
| I4:Most restricted | 11 | 12 | 16 | 27 | 40 |
| I5:Relaxation of restrictions | 24 | 548 | 1071 | 1595 | 2119 |
|  |  |  |  |  |  |
| ***Other*** |  |  |  |  |  |
| I1:Pre-Covid | 13 | 13 | 13 | 13 | 13 |
| I2:First publicized case | 20 | 24 | 29 | 33 | 37 |
| I3:Increasing restrictions | 1 | 19 | 36 | 60 | 85 |
| I4:Most restricted | 53 | 81 | 110 | 139 | 167 |
| I5:Relaxation of restrictions | N/A |  |  |  |  |
|  |  |  |  |  |  |
| ***Unemployed*** |  |  |  |  |  |
| I1:Pre-Covid | 3 | 29 | 123 | 1253 | 31081 |
| I2:First publicized case | 6 | 16 | 57 | 1783 | 13477 |
| I3:Increasing restrictions | 0 | 6 | 18 | 33 | 13466 |
| I4:Most restricted | 0 | 15 | 32 | 53 | 2752 |
| I5:Relaxation of restrictions | 0 | 16 | 25 | 54 | 56060 |

Table S-9. Curfew time RoG (in meters) - by Village

|  | **Minimum** | **Q1** | **Median** | **Q3** | **Maximum** |
| --- | --- | --- | --- | --- | --- |
|  |  |  |  |  |  |
| ***Village A*** |  |  |  |  |  |
| I1:Pre-Covid | 3 | 39 | 929 | 5691 | 22346 |
| I2:First publicized case | 7 | 21 | 90 | 3148 | 15771 |
| I3:Increasing restrictions | 0 | 11 | 18 | 33 | 13466 |
| I4:Most restricted | 1 | 12 | 20 | 34 | 9170 |
| I5:Relaxation of restrictions | 14 | 24 | 38 | 48 | 4737 |
|  |  |  |  |  |  |
| ***Village B*** |  |  |  |  |  |
| I1:Pre-Covid | 10 | 32 | 58 | 237 | 4987 |
| I2:First publicized case | 6 | 17 | 27 | 375 | 4834 |
| I3:Increasing restrictions | 5 | 9 | 20 | 35 | 56 |
| I4:Most restricted | 6 | 33 | 53 | 282 | 3986 |
| I5:Relaxation of restrictions | 17 | 49 | 94 | 2811 | 56060 |
|  |  |  |  |  |  |
| ***Village C*** |  |  |  |  |  |
| I1:Pre-Covid | 6 | 18 | 103 | 780 | 31081 |
| I2:First publicized case | 15 | 20 | 58 | 218 | 3497 |
| I3:Increasing restrictions | 0 | 1 | 15 | 60 | 153 |
| I4:Most restricted | 0 | 15 | 30 | 51 | 251 |
| I5:Relaxation of restrictions | 0 | 4 | 47 | 133 | 1466 |
|  |  |  |  |  |  |
| ***Village D*** |  |  |  |  |  |
| I1:Pre-Covid | 0 | 16 | 57 | 63 | 3034 |
| I2:First publicized case | 9 | 22 | 35 | 48 | 62 |
| I3:Increasing restrictions | 85 | 96 | 107 | 7383 | 14659 |
| I4:Most restricted | 0 | 15 | 25 | 86 | 167 |
| I5:Relaxation of restrictions | 12 | 28 | 94 | 228 | 452 |

Table S-10. Normal time RoG (in meters) - by Sex

|  | **Minimum** | **Q1** | **Median** | **Q3** | **Maximum** |
| --- | --- | --- | --- | --- | --- |
|  |  |  |  |  |  |
| ***Male*** |  |  |  |  |  |
| I1:Pre-Covid | 421 | 777 | 2378 | 5198 | 14565 |
| I2:First publicized case | 21 | 298 | 1034 | 3539 | 15511 |
| I3:Increasing restrictions | 20 | 91 | 241 | 1934 | 19803 |
| I4:Most restricted | 0 | 277 | 2118 | 3231 | 17580 |
| I5:Relaxation of restrictions | 25 | 223 | 1284 | 2910 | 10639 |
|  |  |  |  |  |  |
| ***Female*** |  |  |  |  |  |
| I1:Pre-Covid | 9 | 717 | 2381 | 7933 | 35004 |
| I2:First publicized case | 23 | 459 | 2636 | 5416 | 19945 |
| I3:Increasing restrictions | 0 | 35 | 80 | 2942 | 16553 |
| I4:Most restricted | 7 | 34 | 191 | 2903 | 16480 |
| I5:Relaxation of restrictions | 23 | 172 | 2324 | 4506 | 49540 |

Table S-11. Normal time RoG (in meters) - by Age

|  | **Minimum** | **Q1** | **Median** | **Q3** | **Maximum** |
| --- | --- | --- | --- | --- | --- |
|  |  |  |  |  |  |
| ***<= 35*** |  |  |  |  |  |
| I1:Pre-Covid | 94 | 663 | 2327 | 7477 | 35004 |
| I2:First publicized case | 21 | 388 | 1664 | 3696 | 19945 |
| I3:Increasing restrictions | 0 | 47 | 180 | 4113 | 19803 |
| I4:Most restricted | 7 | 49 | 334 | 3109 | 16480 |
| I5:Relaxation of restrictions | 23 | 118 | 832 | 4741 | 27289 |
|  |  |  |  |  |  |
| ***>35*** |  |  |  |  |  |
| I1:Pre-Covid | 9 | 777 | 2516 | 7800 | 23895 |
| I2:First publicized case | 23 | 337 | 2790 | 5380 | 16361 |
| I3:Increasing restrictions | 3 | 31 | 87 | 2226 | 15770 |
| I4:Most restricted | 0 | 30 | 269 | 3696 | 17580 |
| I5:Relaxation of restrictions | 48 | 1223 | 2809 | 3792 | 49540 |

Table S-12. Normal time RoG (in meters) - by Occupation

|  | **Minimum** | **Q1** | **Median** | **Q3** | **Maximum** |
| --- | --- | --- | --- | --- | --- |
|  |  |  |  |  |  |
| Farmer |  |  |  |  |  |
| I1:Pre-Covid | 323 | 888 | 4415 | 7515 | 12857 |
| I2:First publicized case | 221 | 897 | 2479 | 5971 | 15394 |
| I3:Increasing restrictions | 23 | 28 | 266 | 2552 | 9806 |
| I4:Most restricted | 0 | 52 | 2119 | 6379 | 16480 |
| I5:Relaxation of restrictions | 121 | 1728 | 2324 | 3318 | 27289 |
|  |  |  |  |  |  |
| ***Public health personnel*** |  |  |  |  |  |
| I1:Pre-Covid | 7189 | 9033 | 10877 | 12721 | 14565 |
| I2:First publicized case | 15511 | 15511 | 15511 | 15511 | 15511 |
| I3:Increasing restrictions | 15770 | 15770 | 15770 | 15770 | 15770 |
| I4:Most restricted | 17580 | 17580 | 17580 | 17580 | 17580 |
| I5:Relaxation of restrictions |  |  |  |  |  |
|  |  |  |  |  |  |
| ***Laborer*** |  |  |  |  |  |
| I1:Pre-Covid | 298 | 588 | 1030 | 2902 | 18304 |
| I2:First publicized case | 193 | 553 | 1034 | 2491 | 7615 |
| I3:Increasing restrictions | 53 | 141 | 841 | 6977 | 19803 |
| I4:Most restricted | 28 | 322 | 1972 | 2710 | 8635 |
| I5:Relaxation of restrictions | 84 | 362 | 1307 | 4108 | 10639 |
|  |  |  |  |  |  |
| ***Merchant*** |  |  |  |  |  |
| I1:Pre-Covid | 999 | 1398 | 4708 | 7153 | 15613 |
| I2:First publicized case | 21 | 33 | 1867 | 4576 | 7216 |
| I3:Increasing restrictions | 3 | 13 | 40 | 345 | 11574 |
| I4:Most restricted | 7 | 3094 | 3460 | 4198 | 9309 |
| I5:Relaxation of restrictions | 25 | 3957 | 7889 | 11821 | 15754 |
|  |  |  |  |  |  |
| ***Other*** |  |  |  |  |  |
| I1:Pre-Covid | 94 | 94 | 94 | 94 | 94 |
| I2:First publicized case | 3185 | 7375 | 11565 | 15755 | 19945 |
| I3:Increasing restrictions | 98 | 158 | 219 | 6751 | 13284 |
| I4:Most restricted | 197 | 427 | 657 | 887 | 1117 |
| I5:Relaxation of restrictions | N/A | N/A | N/A | N/A | N/A |
|  |  |  |  |  |  |
| ***Unemployed*** |  |  |  |  |  |
| I1:Pre-Covid | 9 | 797 | 2489 | 9657 | 35004 |
| I2:First publicized case | 23 | 168 | 2605 | 4047 | 16361 |
| I3:Increasing restrictions | 0 | 31 | 59 | 289 | 16553 |
| I4:Most restricted | 16 | 27 | 52 | 269 | 15489 |
| I5:Relaxation of restrictions | 23 | 94 | 1007 | 4368 | 49540 |

Table S-13. Normal time RoG (in meters) - by Village

|  | **Minimum** | **Q1** | **Median** | **Q3** | **Maximum** |
| --- | --- | --- | --- | --- | --- |
|  |  |  |  |  |  |
| ***Village A*** |  |  |  |  |  |
| I1:Pre-Covid | 9 | 777 | 5970 | 9122 | 23895 |
| I2:First publicized case | 21 | 239 | 2429 | 7515 | 16361 |
| I3:Increasing restrictions | 3 | 28 | 67 | 4113 | 16553 |
| I4:Most restricted | 7 | 33 | 71 | 2186 | 17580 |
| I5:Relaxation of restrictions | 23 | 84 | 1040 | 4781 | 15754 |
|  |  |  |  |  |  |
| ***Village B*** |  |  |  |  |  |
| I1:Pre-Covid | 97 | 602 | 1612 | 6097 | 26712 |
| I2:First publicized case | 206 | 831 | 2957 | 3928 | 5435 |
| I3:Increasing restrictions | 58 | 65 | 128 | 440 | 5021 |
| I4:Most restricted | 28 | 693 | 3155 | 3849 | 4198 |
| I5:Relaxation of restrictions | 78 | 1878 | 3646 | 7373 | 49540 |
|  |  |  |  |  |  |
| ***Village C*** |  |  |  |  |  |
| I1:Pre-Covid | 94 | 867 | 1837 | 2584 | 35004 |
| I2:First publicized case | 141 | 388 | 1664 | 4306 | 19945 |
| I3:Increasing restrictions | 0 | 28 | 266 | 2426 | 13284 |
| I4:Most restricted | 14 | 53 | 220 | 6698 | 16480 |
| I5:Relaxation of restrictions | 116 | 410 | 1648 | 2552 | 4230 |
|  |  |  |  |  |  |
| ***Village D*** |  |  |  |  |  |
| I1:Pre-Covid | 118 | 323 | 2994 | 7189 | 8302 |
| I2:First publicized case | 1673 | 2069 | 2466 | 2862 | 3258 |
| I3:Increasing restrictions | 27 | 80 | 1325 | 6865 | 19803 |
| I4:Most restricted | 0 | 10 | 197 | 1972 | 2119 |
| I5:Relaxation of restrictions | 1284 | 1951 | 2248 | 8565 | 27289 |

# Cross-border analysis

Table S-14. The number of cross-border trips from Thailand to Myanmar, the proportion of participants who took cross-border trips, and the median maximum distance of the trips, for each interval.

Note: Short trips are defined as those for which the maximum distance from the border is less than 500 meters and long trips are those greater than 500 meters.

| Interval | Total number of cross-border trips, short trips, and long trips  per participant per day (x 10^-3^) | Percent of participants who took cross-border trips | Median maximum distance of all trips (Q1 - Q3) (meters) |
| --- | --- | --- | --- |
| I1:Pre-Covid | 53.35, 11.06, 42.30 | 59.6 | 636 (169 - 2861) |
| I2:First publicized case | 24.98, 6.40, 18.58 | 38.8 | 4335 (1010 - 39472) |
| I3:Increasing restrictions | 21.12, 18.46, 3.08 | 9.8 | 308 (206 - 425) |
| I4:Most restricted | 19.39, 15.15, 4.24 | 23.6 | 145 (32 - 369) |
| I5: Relaxation of restrictions | 21.82, 15.67, 5.53 | 32.4 | 221 (85 - 436) |

Table S-15. Cross-border trips - by Sex

| **Sex** | **Short Trips per participant per day (x 10^-3^)** | **Long Trips per participant per day (x 10^-3^)** |
| --- | --- | --- |
| ***Male*** |  |  |
| I1:Pre-Covid | 19 | 4 |
| I2:First publicized case | 11 | 3 |
| I3:Increasing restrictions | 12 | 0 |
| I4:Most restricted | 6 | 1 |
| I5:Relaxation of restrictions | 6 | 1 |
|  |  |  |
| ***Female*** |  |  |
| I1:Pre-Covid | 34 | 11 |
| I2:First publicized case | 12 | 8 |
| I3:Increasing restrictions | 8 | 3 |
| I4:Most restricted | 10 | 3 |
| I5:Relaxation of restrictions | 15 | 6 |

Table S-16. Cross-border trips - by Age

| **Age** | **Short Trips per participant per day (x 10^-3^)** | **Long Trips per participant per day (x 10^-3^)** |
| --- | --- | --- |
| ***<= 35*** |  |  |
| I1:Pre-Covid | 40 | 10 |
| I2:First publicized case | 15 | 6 |
| I3:Increasing restrictions | 17 | 3 |
| I4:Most restricted | 11 | 4 |
| I5:Relaxation of restrictions | 9 | 6 |
|  |  |  |
| ***>35*** |  |  |
| I1:Pre-Covid | 12 | 5 |
| I2:First publicized case | 8 | 6 |
| I3:Increasing restrictions | 3 | 0 |
| I4:Most restricted | 6 | 0 |
| I5:Relaxation of restrictions | 12 | 1 |

Table S-17. Cross-border trips - by Occupation

| **Occupation** | **Short Trips per participant per day (x 10^-3^)** | **Long Trips per participant per day (x 10^-3^)** |
| --- | --- | --- |
| ***Farmer*** |  |  |
| I1:Pre-Covid | 11 | 6 |
| I2:First publicized case | 2 | 4 |
| I3:Increasing restrictions | 0 | 3 |
| I4:Most restricted | 1 | 2 |
| I5:Relaxation of restrictions | 3 | 2 |
|  |  |  |
| ***Public health personnel*** |  |  |
| I1:Pre-Covid | 2 | 0 |
| I2:First publicized case | 3 | 1 |
| I3:Increasing restrictions | 0 | 0 |
| I4:Most restricted | 1 | 0 |
| I5:Relaxation of restrictions | 0 | 0 |
|  |  |  |
| ***Laborer*** |  |  |
| I1:Pre-Covid | 22 | 2 |
| I2:First publicized case | 9 | 2 |
| I3:Increasing restrictions | 5 | 0 |
| I4:Most restricted | 7 | 0 |
| I5:Relaxation of restrictions | 7 | 2 |
|  |  |  |
| ***Merchant*** |  |  |
| I1:Pre-Covid | 2 | 2 |
| I2:First publicized case | 1 | 1 |
| I3:Increasing restrictions | 0 | 0 |
| I4:Most restricted | 1 | 0 |
| I5:Relaxation of restrictions | 0 | 0 |
|  |  |  |
| ***Other*** |  |  |
| I1:Pre-Covid | 1 | 0 |
| I2:First publicized case | 1 | 0 |
| I3:Increasing restrictions | 0 | 0 |
| I4:Most restricted | 0 | 0 |
| I5:Relaxation of restrictions | 0 | 0 |
|  |  |  |
| ***Unemployed*** |  |  |
| I1:Pre-Covid | 15 | 4 |
| I2:First publicized case | 5 | 4 |
| I3:Increasing restrictions | 5 | 0 |
| I4:Most restricted | 6 | 1 |
| I5:Relaxation of restrictions | 11 | 3 |

Table S-18. Cross-border trips - by Village

| **Village** | **Short Trips per participant per day (x 10^-3^)** | **Long Trips per participant per day (x 10^-3^)** |
| --- | --- | --- |
| ***Village A*** |  |  |
| I1:Pre-Covid | 31 | 5 |
| I2:First publicized case | 15 | 7 |
| I3:Increasing restrictions | 5 | 0 |
| I4:Most restricted | 10 | 0 |
| I5:Relaxation of restrictions | 9 | 1 |
|  |  |  |
| ***Village B*** |  |  |
| I1:Pre-Covid | 8 | 5 |
| I2:First publicized case | 4 | 2 |
| I3:Increasing restrictions | 5 | 0 |
| I4:Most restricted | 6 | 1 |
| I5:Relaxation of restrictions | 9 | 2 |
|  |  |  |
| ***Village C*** |  |  |
| I1:Pre-Covid | 13 | 5 |
| I2:First publicized case | 3 | 2 |
| I3:Increasing restrictions | 0 | 3 |
| I4:Most restricted | 1 | 3 |
| I5:Relaxation of restrictions | 2 | 4 |
|  |  |  |
| ***Village D*** |  |  |
| I1:Pre-Covid | 0 | 0 |
| I2:First publicized case | 2 | 1 |
| I3:Increasing restrictions | 11 | 0 |
| I4:Most restricted | 0 | 0 |
| I5:Relaxation of restrictions | 1 | 0 |

# Village boundary mobility analysis

Table S-19. Percentage time spent outside the home village and number of long and short trips.

Note: Short trips are defined as those for which the maximum distance from the village boundary is less than 500 meters and long trips are those greater than 500 meters.

|  | **Percent time spent outside home village (SD)** | **Short Trips per participant per day (SD)** | **Long Trips per participant per day (SD)** |
| --- | --- | --- | --- |
| I1:Pre-Covid | 9 (9) | 3 (8) | 5 (12) |
| I2:First publicized case | 12 (17) | 3 (11) | 3 (9) |
| I3:Increasing restrictions | 24 (39) | 3 (10) | 1 (3) |
| I4:Most restricted | 13 (27) | 2 (9) | 1 (5) |
| I5:Relaxation of restrictions | 14 (24) | 1 (3) | 2 (5) |

Table S-20. Village boundary mobility analysis - by Village

|  | **Percent time spent outside home village (SD)** | **Short Trips per participant per day (SD)** | **Long Trips per participant per day (SD)** |
| --- | --- | --- | --- |
| ***Village A*** |  |  |  |
| I1:Pre-Covid | 9 (9) | 2 (10) | 3 (15) |
| I2:First publicized case | 10 (20) | 1 (7) | 1 (10) |
| I3:Increasing restrictions | 27 (42) | 0 (1) | 1 (4) |
| I4:Most restricted | 3 (9) | 1 (9) | 1 (6) |
| I5:Relaxation of restrictions | 8 (24) | 0 (3) | 1 (4) |
|  |  |  |  |
| ***Village B*** |  |  |  |
| I1:Pre-Covid | 10 (11) | 1 (7) | 0 (4) |
| I2:First publicized case | 19 (16) | 1 (14) | 1 (8) |
| I3:Increasing restrictions | 7 (10) | 2 (12) | 0 (1) |
| I4:Most restricted | 11 (11) | 1 (11) | 0 (4) |
| I5:Relaxation of restrictions | 30 (31) | 1 (3) | 1 (6) |
|  |  |  |  |
| ***Village C*** |  |  |  |
| I1:Pre-Covid | 9 (6) | 1 (7) | 1 (9) |
| I2:First publicized case | 13 (13) | 1 (12) | 1 (8) |
| I3:Increasing restrictions | 17 (29) | 0 (0) | 0 (4) |
| I4:Most restricted | 15 (29) | 0 (0) | 0 (4) |
| I5:Relaxation of restrictions | 16 (15) | 0 (1) | 0 (2) |
|  |  |  |  |
| ***Village D*** |  |  |  |
| I1:Pre-Covid | 8 (7) | 0 (1) | 1 (12) |
| I2:First publicized case | 11 (0) | 0 (1) | 0 (4) |
| I3:Increasing restrictions | 0 (3) | 0 (0) | 0 (1) |
| I4:Most restricted | 1 (1) | 0 (0) | 0 (2) |
| I5:Relaxation of restrictions | 11 (11) | 0 (1) | 0 (1) |
