# Supplementary figures and images for: Effects of COVID-19 government travel restrictions on mobility in a rural border area of Northern Thailand: A mobile phone tracking study
Source: PLoS One. 2021 Feb 3;16(2):e0245842. doi: 10.1371/journal.pone.0245842 (PMC7857734; doi:10.1371/journal.pone.0245842)

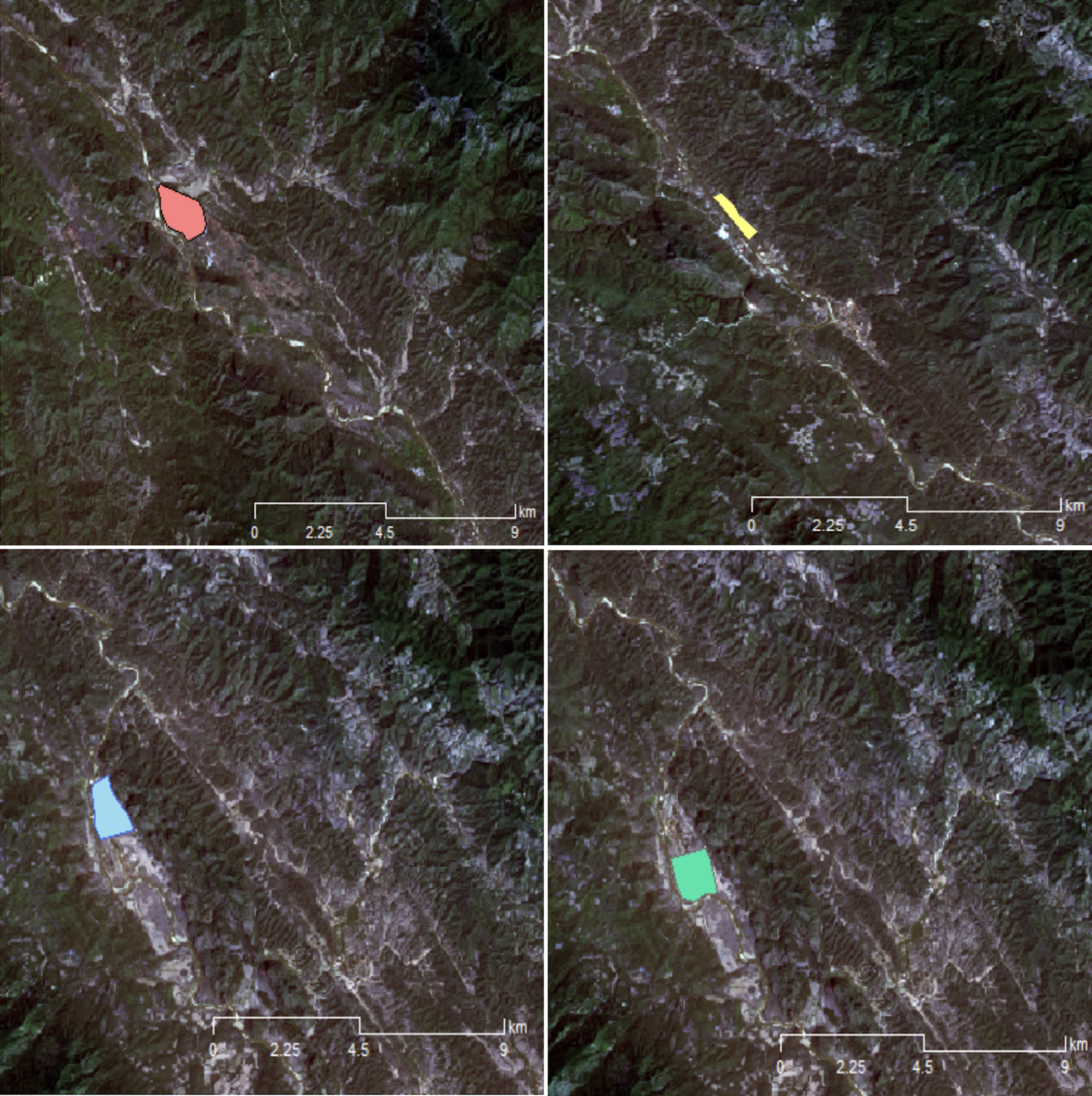

Supplement: S1 Fig — (TIF) [file pone.0245842.s002.tif]
